# Risk of infections in patients with NAFLD and Type 2 Diabetes under treatment with SGLT2 inhibitors and relationship with liver outcomes: A retrospective case-control study

**DOI:** 10.3389/fendo.2022.945626

**Published:** 2022-08-24

**Authors:** Juan Bañares, Ramiro Manzano-Nuñez, Alba Prió, Jesús Rivera-Esteban, Laura Camps-Relats, Ana Villarejo, Lourdes Ruiz-Ortega, Mònica Pons, Andreea Ciudin, María Teresa Salcedo, Víctor Vargas, Joan Genescà, Juan M. Pericàs

**Affiliations:** ^1^ Liver Unit, Internal Medicine Department, Hospital Universitari Vall d’Hebron, Vall d’Hebron Institut de Recerca (VHIR), Vall d’Hebron Barcelona Campus Hospitalari, Barcelona, Spain; ^2^ Medicine Department, Universitat Autònoma de Barcelona, Barcelona, Spain; ^3^ Centro de Investigación Biomédica en Red de enfermedades digestivas y hepáticas (CIBERehd), Madrid, Instituto de Salud Carlos III, Madrid, Spain; ^4^ Endocrinology and Nutrition Department, Hospital Universitari Vall d’Hebron, Vall d’Hebron Institut de Recerca (VHIR), Vall d’Hebron Barcelona Campus Hospitalari, Barcelona, Spain; ^5^ CIBER de Diabetes y Enfermedades Metabólicas Asociadas (CIBERDem), Instituto de Salud Carlos III, Madrid, Spain; ^6^ Pathology Department, Hospital Universitari Vall d’Hebron, Vall d’Hebron Barcelona Campus Hospitalari, Barcelona, Spain

**Keywords:** NAFLD, type 2 diabetes mellitus, sodium-glucose co-transporter-2 inhibitors, infections, hepatic outcomes

## Abstract

**Introduction:**

Non-alcoholic fatty liver disease (NAFLD) is the most prevalent chronic liver disease in developed countries, with its incidence growing parallel to the epidemics of obesity and type 2 diabetes mellitus (T2DM). Sodium-glucose co-transporter-2 inhibitors (SGLT2i) are becoming a cornerstone in the management of cardiovascular health and some studies suggest the potential role in NAFLD. However, patients under treatment with SGLT2i are at risk of developing genitourinary fungal infections (GFIs). Moreover, both NAFLD and SGLT2i have a strong influence on the immune system, and therefore the risk of infections other than GFIs could be increased in NAFLD patients treated with SGLT2i. We aimed to examine the possible association of SGLT2i with infections and hepatic outcomes in NAFLD patients.

**Methods:**

We conducted a case-control study including NAFLD patients with T2DM visited at the Liver Unit outpatient clinic from 2016 to 2021 with a minimum follow-up of 6 months by selecting 65 patients receiving SGLT2i and 130 matched patients with other types of antidiabetic treatment.

**Results:**

During follow-up, GFIs were significantly higher in the SGLT2i group (15.4% vs. 3.8%; p=0.008), whereas there were no differences in the occurrence of overall infections (41.5% vs. 30%; p=0.1) nor in other types of specific infections. In the multivariable analysis, treatment with SGLT2i was not independently associated with higher odds of overall infection. On the other hand, SGLT2i patients showed a significantly lower incidence of hepatic events (1.5% vs. 10.7%; p=0.02). There were no significant different in all-cause mortality between cases and controls.

**Conclusions:**

NAFLD patients with T2DM receiving SGLT2i more frequently presented GFIs, whereas the incidence of other types of infections was not found to be higher than in other patients with NAFLD and T2DM treated with other drugs. Moreover, SGLT2i-treated patients had a lower occurrence of hepatic events. Further studies are warranted to validate our data.

## Introduction

Non-alcoholic fatty liver disease (NAFLD) encompasses a progressive clinical spectrum: from simple steatosis, through inflammation [i.e., non-alcoholic steatohepatitis (NASH)] and fibrosis, to liver cirrhosis (1, 2). From an epidemiological standpoint, parallel to the growing epidemic of obesity and type 2 diabetes mellitus (T2DM), NAFLD has emerged as the most prevalent liver disease in the US and probably will become the leading cause of liver transplantation in the upcoming years (3). It is estimated that 25% of general population in Western countries to have NAFLD, but this prevalence increases up to 60-80% in patients with obesity or T2DM and can reach 80-100% when both risk factors are present (2, 3). In addition, it is estimated that 20-30% of patients with NAFLD will progress to liver inflammation and fibrosis (2–4).

NAFLD is considered the liver manifestation of metabolic syndrome, both sharing multiple pathophysiological mechanisms such as insulin resistance (1–3). Moreover, improvements in metabolic factors such as weight loss, are associated with amelioration in inflammation and liver fibrosis (1). Sodium-glucose co-transporter-2 inhibitors (SGLT2i) are now solidly established amongst the armamentarium to improve metabolic status and cardiovascular health in T2DM, chronic kidney disease and cardiovascular diseases (5, 6). Emerging data suggest that SGLT2i may play a role in treating NAFLD being associated with an impact in the metabolic status, including reduction in liver fat content and even histologic improvement in liver steatosis and fibrosis (7–10). Such effects could have a clinically relevant impact on the outcomes of patients with T2DM and NAFLD, making SGLT2i an attractive therapeutic alternative. Even though there are currently several clinical trials underway assessing their efficacy to treat NAFLD (11, 12), there is insufficient information regarding an impact in clinical practice.

SGLT2i inhibit glucose reabsorption in the kidney *via* inhibition of the SGLT channels primarily located in the proximal tubules, promoting glycosuria, which has been associated with a higher incidence of urinary and genital infections, mainly caused by fungi, with odds ratios ranging approximately from 3 to 5 (13–17). Moreover, patients with T2DM have increased susceptibility to a wide array of infections due to variable degrees of baseline immunosuppression caused by complex mechanisms that are tightly intertwined with pathways leading to the enhanced systemic inflammation and immune system dysfunction characteristic of advanced liver disease, while obesity and NAFLD may also increase infection susceptibility (1, 18, 19). However, no studies have investigated whether T2DM patients with NAFLD treated with SGLT2i present increased rates of infections and particularly genitourinary fungal infections (GFIs).

The present study aimed to examine the impact of SGLT2i treatment in the incidence of infections in patients with NAFLD and T2DM. In addition, we aimed to investigate whether NAFLD patients treated with SGLT2i presented significant differences in liver outcomes compared to those not receiving SGLT2i.

## Methods

### Design and setting

Case-control study conducted at the Vall d’Hebron University Hospital (VHUH), a tertiary care setting with 1,300 beds in Barcelona, Spain.

### Participants

**Figure 1:**
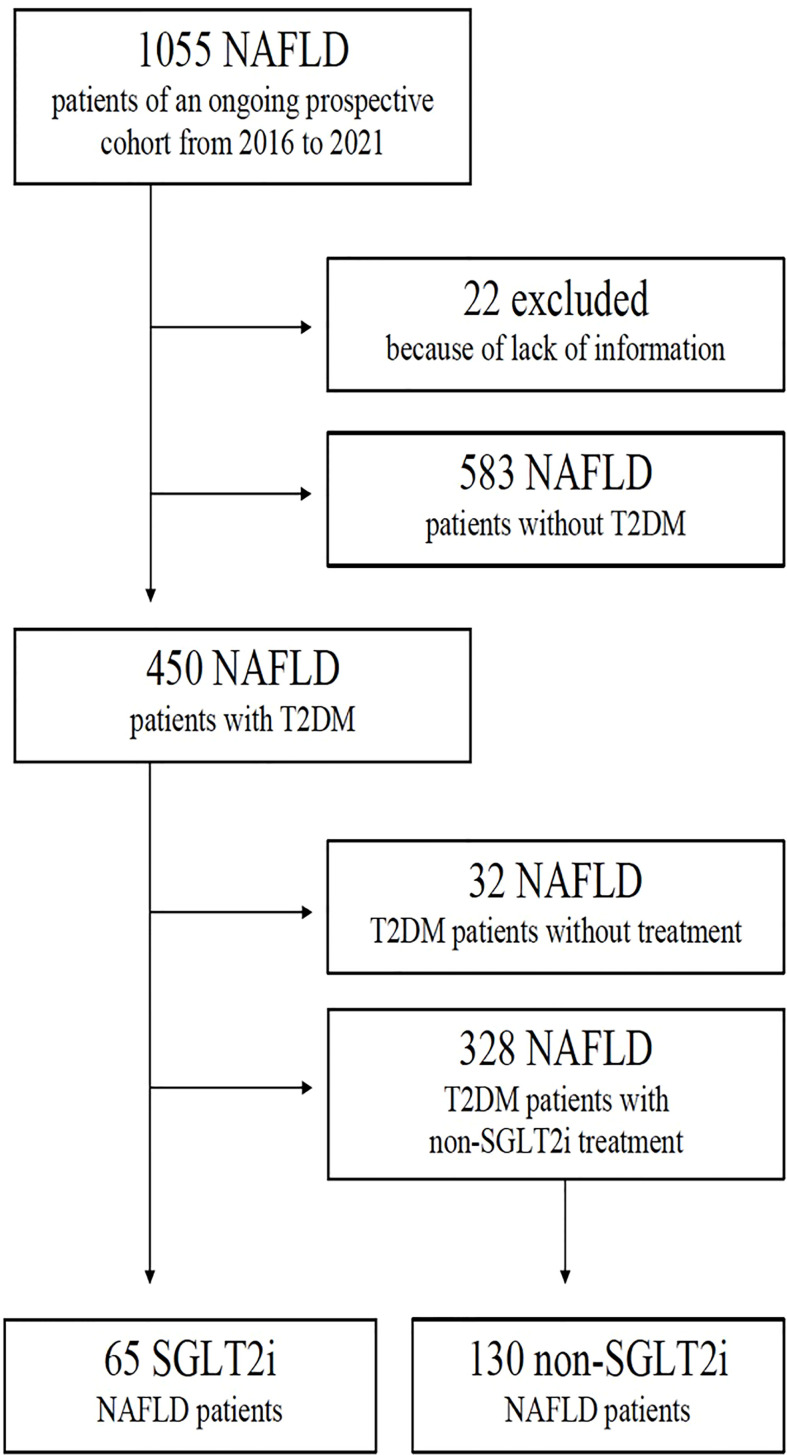
Patients’ disposition flowchart.

An ongoing prospective cohort study on NAFLD was used to identify patients considered eligible to be included in the present case-control study. The ongoing prospective cohort study includes consecutive patients from the VHUH NAFLD outpatient clinics diagnosed with NAFLD and T2DM from 2016 to 2021. Patients with a diagnosis of both NAFLD and T2DM were considered eligible for inclusion. Patients with NAFLD and T2DM receiving treatment with SGLT2i and with a minimum of 6 months of follow-up were identified and defined as cases. NAFLD and T2DM patients receiving other antidiabetic drug different than SGLT2i were considered as potential controls (see flowchart in **Figure 1**). Matching variables were sex and age (+/- 3 years).

We excluded patients with Child B and C cirrhosis from the analysis to avoid bias in the allocation of SGLT2i treatment, as we considered that patients with more severe liver disease were less likely to receive treatment with SGLT2i because these are not widely recommended in patients with moderate-severe hepatic insufficiency, and also because patients with advanced chronic liver disease are at higher risk of infections and these might trigger hepatic events.

### Outcomes

The occurrence of any type of infection was considered as the primary outcome of interest. Secondary outcomes included the occurrence of each type of infection separately, the occurrence of hepatic events (only the first episode accounted for each patient), and all-cause mortality.

### Definitions

The diagnostic criteria for NAFLD and T2DM are described elsewhere (1, 20). Briefly, NAFLD was diagnosed either based on steatosis in abdominal ultrasound or transient elastography and the presence of at least one feature of metabolic syndrome or by liver biopsy. T2DM was diagnosed following current clinical practice recommendations from the American Diabetes Association (20). Cirrhosis was defined by histological analysis when available and/or by a combination of clinical, analytical, liver stiffness measurement, endoscopic assessment and/or radiological evidence of advanced chronic liver disease. Hepatic events were defined as the presence of ascites, hepatic encephalopathy, and upper gastrointestinal bleeding. Infections were defined as urogenital fungal infections, urogenital bacterial infections, bacteremia, upper and lower respiratory tract infections, COVID-19, intra-abdominal, skin and soft tissues infections, and spontaneous bacterial peritonitis. Infections were diagnosed in a real clinical setting by means of clinical presentation and, when needed, radiological, laboratory and microbiological analysis.

### Ethics

The study protocol was approved by the VHUH Ethics Committee for Clinical Research (protocol code: PR(AG)222/2021) and conformed to the ethical guidelines of the 1975 Declaration of Helsinki.

### Statistical analyses

Data were collected into a pre-specified and de-identified database in electronic format. Categorical variables were presented as frequencies and percentages. Continuous variables were expressed as means with standard deviation or medians with interquartile ranges as appropriate according to data distribution. Categorical variables were compared with Chi square test or Fisher exact test where appropriate. Continuous variables were compared using T test or nonparametric tests when necessary. A multivariate logistic regression analysis adjusted by relevant covariates was used to examine the association between SGLT2i use and the occurrence of infections. Findings were presented as odds ratios (OR) with 95% confidence intervals. Kaplan-Meier survival curves and long-rank test were calculated for the occurrence of overall infections and hepatic events along time. All analyses were performed in STATA statistical software.

## Results

### Patients

The flowchart of patients’ disposition is shown in **Figure 1**.

After applying the inclusion and exclusion criteria, sixty-five patients with NAFLD and T2DM received SGLT2i and were considered as cases, whereas 130 diabetic patients with NAFLD not receiving SGLT2i were matched as controls. Characteristics of patients are shown in **Table 1**. Overall median follow-up was 34.4 months (IQR 17-48.3), with no significant differences found between cases and controls. There were neither differences regarding age, sex (matching variables), the prevalence of comorbidities, or the values of glycosylated hemoglobin, albumin, and liver enzymes between groups. Patients receiving SGLT2i had significantly higher BMI values and were more likely to present obesity.

**Table 1:** Demographics and clinical characteristics of cases (SGLT2i users) and controls (non-SGLT2i users) in a cohort of patients with NAFLD and type 2 diabetes.

	Total (n = 195)	SGLT2i users (n = 65)	Non-SGLT2i users (n = 130)	p-value
Follow-up, median months (IQR)	34.4 (17-48.3)	34.4 (16.5-48.2)	34.4 (17.1-49)	0.5
Age, median (IQR)	62 (57-68)	61 (57-66)	63 (57-68)	0.3
≥70 years, n (%)	40 (20.5)	10 (15.4)	30 (23)	0.2
Male sex, n (%)	104 (53.3)	35 (53.8)	69 (53.1)	0.9
**Comorbidities**				
Body Mass Index, median (IQR)	31.5 (28-34)	33.5 (29.7-36.2)	30.4 (27.3-34.2)	0.001
Obesity, n (%)	116 (61.4)	47 (74.6)	69 (54.7)	0.008*
Hypertension, n (%)	145 (74.3)	51 (78.5)	94 (72.3)	0.3
Dyslipidemia, n (%)	152 (78.0)	55 (84.6)	97 (74.6)	0.1
Ischemic Heart Disease, n (%)	18 (9.2%)	5 (7.8%)	13 (10)	0.7
Heart Failure, n (%)	17 (8.7)	7 (10.7)	10 (7.7)	0.4
COPD, n (%)	24 (12.3)	12 (18.4)	12 (9.2)	0.06
CKD, n (%)	20 (10.2)	6 (9.2)	14 (10.7)	0.7
Child A cirrhosis, n (%)	65 (33.7)	21 (33.3)	44 (33.8)	0.9
**SGLT2i**				
Canagliflozin, n (%)	–	9 (13.9)	–	
Dapagliflozin, n (%)	–	31 (47.7)	–	
Empagliflozin, n (%)	–	35 (35.4)	–	
Other/No data, n (%)	–	2 (3.1)	–	
**Concomitant T2DM treatment**				
Insulin, n (%)	76 (39.0)	32 (49.2)	44 (33.9)	0.03
Metformin, n (%)	161 (82.6)	53 (81.5)	108 (83.1)	0.7
DDP-4 inhibitors, n (%)	53 (27.2)	18 (27.7)	35 (26.9)	0.9
GLP-1 receptor agonists, n (%)	50 (25.6)	26 (40.0)	24 (18.5)	0.001
Pioglitazone, n (%)	35 (18.0)	12 (18.5)	23 (17.7)	0.9
Sulfonylureas, n (%)	4 (2.1)	2 (3.1)	2 (1.5)	0.5
Metaglinides, n (%)	5 (2.6)	2 (3.1)	3 (2.3)	0.8
**Number of antidiabetic drugs**				
Monotherapy	49 (25.1)	0 (0.0)	49 (37.7)	<0.001
2-drugs regime	68 (34.9)	13 (20.0)	55 (42.3)	0.002
3-drugs regime	52 (26.7)	30 (46.2)	22 (16.9)	<0.001
4-drugs regime	19 (9.7)	16 (24.6)	3 (2.3)	<0.001
5-drugs regime	7 (3.6)	6 (9.2)	1 (0.8)	0.002
**Lipid-lowering agents**				
Statins^†^	110 (56.4)	43 (66.2)	67 (51.5)	0.05
Fibrates^†^	35 (17.9)	18 (27.7)	17 (13.1)	0.1
Ezetimibe^†^	12 (6.2)	2 (3.1)	10 (7.7)	0.2
Other/No data	4 (2.1)	0 (0.0)	4 (3.1)	0.146
No treatment	58 (29.7)	13 (20.0)	45 (34.6)	0.03
**Baseline Blood Tests**				
HbA1c, median (QIR)	7.25 (6.7-8)	7.5 (6.9-8.5)	7 (6.5-8)	0.01
Glucose, median (IQR)	137 (110-167)	141 (114-167)	133 (108-165)	0.1
Platelets, median (IQR)	224 (161-273)	232 (183-271)	215 (152-274)	0.1
Creatinine, median (IQR)	0.79 (0.63-0.93)	0.8 (0.63-0.95)	0.77 (0.62-0.92)	0.5
Albumin, median (IQR)	4.3 (4.1-4.5)	4.3 (4.1-4.5)	4.3 (4.1-4.5)	0.5
INR, median (IQR)	0.97 (0.92-1.04)	0.96 (0.93-1.02)	0.98 (0.92-1.04)	0.6
AST (GOT), median (IQR)	36 (24-52)	35 (24-52)	36 (24-51)	0.8
ALT (GPT), median (IQR)	40 (26-56)	39 (25-56)	40 (27-60)	0.6
GGT, median (IQR)	72 (37-137)	67 (34-132)	72 (38-147)	0.2
Alkaline phosphatase, median (IQR)	85 (69-114)	81 (62-100)	89 (72-123)	0.01*
FIB-4, median (IQR)	1.66 (1.16-2.9)	1.56 (1.16-2.6)	1.68 (1.16-3.17)	0.3
LSM, median kPa (IQR)				
Overall	10 (6.8-18.3)	10.1 (7.7-16.6)	10 (6.4-18.6)	0.6
Cirrhotics	19.8 (15.6-34.9)	19.8 (15-27)	20.4 (17-43.2)	0.4

^†^Lipid-lowering agents alone or in combination with other drug classes.

ALT, alanine transferase; AST, aspartate transferase; GGT, gamma glutamyl transferase; CKD, chronic kidney disease; COPD, chronic obstructive pulmonary disease; HbA1c, glycosylated hemoglobin; LSM, liver stiffness measurement; SGLT2i, sodium-glucose co-transporter-2 inhibitors. * means statistical significance at, p < 0.05.

### Infections

Outcomes’ information is outlined in **Table 2**. There were no significant differences in the occurrence of overall infections between groups (41.5% vs. 30%; p=0.1). With respect to the infection’s etiology, the proportion of patients presenting genital mycotic infections was significantly higher in patients receiving SGLT2i compared to those not receiving SGLT2i (15.4% vs. 3.8%; p=0.008), with incidence rates of 5.12 and 1.28 cases per 100 person-year in SGLT2i and non-SGLT2i groups, respectively (p<0.001). There were no significant differences in the proportion of the other types of infections between the two group of patients, including urinary tract infections of bacterial etiology (10.7% vs. 7.7%, p=0.4).

**Table 2:** Outcomes in cases (SGLT2i users) and controls (non- SGLT2i users) in a cohort of patients with NAFLD and type 2 diabetes.

	Total (n = 195)	SGLT2i users (n = 65)	Non-SGLT2i (n = 130)	p-value
Overall infection (total), n (%)	66 (39)	27 (41.5)	39 (30)	0.1
Respiratory tract infection, n (%)	16 (8.2)	5 (7.7)	11 (8.4)	0.9
Abdominal infection, n (%)	4 (2)	2 (3.1)	2 (1.5)	0.6
Bacterial UT infection, n (%)	17 (8.7)	7 (10.7)	10 (7.7)	0.4
Mycotic UT/genital infection, n (%)	15 (7.7)	10 (15.4)	5 (3.8)	0.008
Skin and Soft tissue infection, n (%)	4 (2)	1 (1.5)	3 (2.3)	1
Bacteremia, n (%)	4 (2)	1 (1.5)	3 (2.3)	1
COVID-19, n (%)	2 (1)	1 (1.5)	1 (1)	1
SBP, n (%)	1 (1.9)	0 (0)	1 (2.5)	1
Hepatic Events, n (%)	15 (7.7)	1 (1.5)	14 (10.7)	0.02
Ascites, n (%)	10 (5.1)	1 (1.5)	9 (6.2)	0.1
HE, n (%)	6 (3.1)	0 (0)	6 (4.6)	0.1
UGB, n (%)	7 (13.2)	0 (0)	5 (17.5)	0.1
Mortality, n (%)	10 (5.1)	1 (1.5)	9 (6.9)	0.1

SGLT2i, Sodium-glucose co-transporter-2 inhibitors; UT, urinary tract; COVID-19, Coronavirus disease 2019; SBP, Spontaneous bacterial peritonitis; HE, hepatic encephalopathy UGB, upper gastrointestinal tract bleeding.

As shown in **Table 3**, multivariate logistic regression analysis showed no significant risk-adjusted odds of infections with the use of SGLT2i (OR 1.68, 95% CI 0.93-3.05; p=0.084).

**Table 3:** Multivariate logistic regression analysis for risk of infection.

Variable	Adjusted HR (95% CI)	p-value
SGLT2i	1.68 (0.93-3.05)	0.084
HbA1c	1.09 (0.87-1.36)	0.43
Obesity^†^	1.35 (0.70-2.59)	0.36
Platelets	1.0 (0.99-1.004)	0.39
Age	1.02 (0.98-1.07)	0.17
Sex (males)	0.95 (0.52.1.74)	0.89

HbA1c, glycosylated hemoglobin; SGLT2i, Sodium-glucose co-transporter-2 inhibitors ^†^(BMI >30 kg/m^2^).

**Figure 2:**
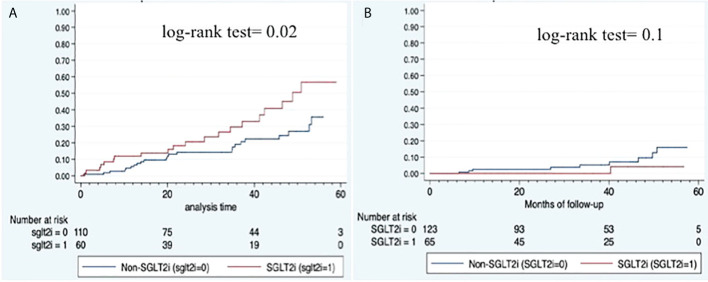
Kaplan-Meier survival curves. **(A)** Overall infections; **(B)** Hepatic events.

Kaplan-Meier survival curve on the occurrence of overall infections is shown in **Figure 2A** (log-rank test= 0.02).

### Hepatic events

Overall, patient who received SGLT2i showed a significantly lower incidence of hepatic events (1.5% vs. 10.7%; p=0.02) although the proportion of cirrhosis was similar between groups. Each individual decompensating event was less frequent in SGLT2i treated patients (**Table 2**) although the difference did not reach statistical significance. **Figure 2B** shows the Kaplan-Meier curve for hepatic events (log-rank test= 0.1).

### Mortality

No significant differences in overall mortality [1.5% vs 6.9%; p=0.1] were observed between groups. Out-of-hospital cardiac arrest was the cause of death of the only patient who died in the SGLT2i group. In the non-SGLT2i group 5 patients died because of an infection (two of them of COVID-19) and one as a direct complication of hepatocellular carcinoma. The other three died of cardiac arrest, extrahepatic cancer and a motor vehicle collision.

## Discussion

In this study we aimed at examining the frequency of infections and hepatic events in patients with NAFLD and T2DM treated with SGLT2i as compared to patients treated with other antidiabetic agents. Although we did not find a significantly higher rate of overall infections in patients treated with SGLT2i, we described for the first time that genitourinary fungal infections are also more frequent in T2DM patients with NAFLD treated with SGLT2i. Moreover, we found that SGLT2i-treated patients had lower rates of hepatic events during the follow-up time included in the study.

Despite there were no significant differences in the occurrence of overall infectious episodes among SGLT2i patients, these were more likely to present genitourinary fungal infections (GFIs). In the general population, the incidence of GFIs has been estimated to be approximately 1-2% (13). As previously reported across a large body of literature, through their glycosuric effect there is a higher risk of these types on infections in SGLT2i users, with rates between 3 to 8% (13, 15, 16). Several randomized controlled trials revealed a 3- to 4-fold increase for SGLT2i compared with placebo (14, 17), which led manufacturers to include GFIs as common adverse reactions in the prescribing information of SGLT2i. Furthermore, observational real-world studies analyzing claims data revealed similar results. For example, a large population-based observational study, analyzing data of more than 40,000 patients, found a two-fold increase in the risk of GFIs with the use of SGLT2i in older diabetic patients (16). Our study is the first to corroborate these findings in a NAFLD cohort. Furthermore, the 15% rate of GFIs in NAFLD patients found in our cohort is higher than those previously reported in SGLT2i-treated diabetic patients without NAFLD (17), which could point to a synergistic effect of NAFLD-related underlying immune dysfunction (1) added to the SGLT2i intrinsic increased risk of these infections. NAFLD indeed has been linked to immune system malfunctioning (18), including microcirculation disarrangements leading to diminished microbial clearance, undermined function of neutrophils and natural killer cells, and deficiency of vitamin-D levels, which in turn further impair innate immunity (19).

Notwithstanding the increased risk of GFIs infection, treatment with SGLT2i was not associated with and overall increase of infections. In contrast, HbA1c levels were independently associated with the risk of infections. This has been previously described in patients with T2DM (21–23). To our knowledge, this is the first study that corroborates this finding in NAFLD patients. As infections can precipitate acute decompensation and acute-on-chronic liver failure in cirrhotic patients (24), improving glycemic control could reduce the risk of infection. Further studies are required to better understand the pathologic pathways and clinical implications of these findings.

We found that patients with T2DM and comorbid NAFLD receiving treatment with SGLT2i were less likely to present hepatic events. Though preliminary, these data suggest the potential beneficial effects of SGLT2i in avoiding liver disease-related complications amongst patients with T2DM and NAFLD. Translational research in animal models and clinical studies has shown that SLGT2i use is related to improved hepatic metabolism (9). Its use has been associated with reductions in hepatic steatosis measured by controlled attenuation parameter, MRI-proton density fat fraction or magnetic resonance spectroscopy (7), as well as liver stiffness ameliorations in patients with previous significant fibrosis (8). In a single-arm pilot study with paired biopsies demonstrated that SGLT2i might induce histological improvements in NASH features without a worsening of fibrosis (10, 25). Moreover, a small randomized controlled trial found that SGLT2i treatment resulted in reduced liver fat content by MRI-derived proton density fat fraction and improvements in liver enzymes, compared to placebo (12). Several ongoing clinical trials are currently assessing the effects of SGLT2i in patients with NAFLD and NASH, which may provide more definitive answers. Parallel to their well-known beneficial cardiovascular and renal effects (5), SLGT2i may exert additional hepatic protection, which could be a cornerstone in the treatment of T2DM patients with NAFLD. Moreover, while SLGT2i undoubtedly increase GFIs, by improving the metabolic control the overall risk of infection could decrease. This balancing situation presents a trade-off for physicians and patients between metabolic control and adverse effects. Cirrhotic patients, especially those that have already presented decompensations, are of special interest regarding the role of SGLT2i because their combined glycosuric and natriuric effects might be beneficial in patients with hypervolemia and renal dysfunction such as those with hepatorrenal syndrome (26). Nonetheless, SGLT2i are seldom prescribed in patients with advanced liver disease. Preliminary findings by Saffo, Garcia-Tsao and Tadei in a cohort of seventy-eight patients with cirrhosis treated with SGLT2i, including 39 (50%) with non-alcoholic steatohepatitis and 63 (81%) with compensated disease, rates of hepatic decompensation and mortality were not increased compared to most published cohorts of cirrhotics, and SGLT2i use was not identified as a cause of decompensation (27). Whether administering SGLT2i before first decompensation or as earlier as possible in both diabetic and non-diabetic patients might be not only safe but beneficial as suggested by our findings warrants further investigation.

## Limitations

This study is not without limitations and results should be interpreted in the context of the study design. First, we performed a case-control study, which is prone to selection bias thereby limiting the validity of the results presented. In the case of our study, cases had higher rates of obesity, were using more drugs for T2DM, had more insulin use and also statin, and had also higher glycated hemoglobin, which might contribute to both infections and hepatic events. Second, the collection of relevant data was retrospective. Third, the relatively small sample size constrains the robustness of multivariate analyses. Fourth, the potential effects of medications other than SGLT2i (e.g., GLP1 receptor agonists) were not addressed in our analysis. Despite our limitations, we provide data about infectious and metabolic outcomes that are relevant to clinical practice. Nevertheless, these results should be interpreted in the context of hypothesis generating research and should be tested in subsequent, larger, studies.

## Conclusion

When compared with patients with NAFLD and T2DM without SGLT2i treatment, patients receiving SGLT2i were more likely to present genitourinary fungal infections but not other types of infections. In addition, patients receiving SGLT2i had a lower occurrence of hepatic events. Further studies are required to confirm these results.

## Data availability statement

The raw data supporting the conclusions of this article will be made available by the authors, without undue reservation.

## Ethics statement

The studies involving human participants were reviewed and approved by Vall d’Hebron University Hospital Ethics Committee. The patients/participants provided their written informed consent to participate in this study.

## Author contributions

Conceptualization and design: JB, JP; Data collection: JB, AP, JR-E, LC-R, AV, LR-O, MS; Data analysis: RM-N, MP; Interpretation: JR-E, AC, MS, VV, JG, JP; First manuscript drafting: JB, RM-N; Revision and acceptance of last version: All authors; Supervision: JP. All authors contributed to the article and approved the submitted version.

## Acknowledgments

Preliminary analyses related to the present study were presented during the 46^th^ Spanish Conference of the Association for the Study of the Liver (AEEH, Abstract 01213, June 14-16, 2021, Madrid, Spain).

## Conflict of interest 

JP reports having received consulting fees from Boehringer Ingelheim and Novo Nordisk. He has received speaking fees from Gilead, and travel expenses from Gilead, Rubió, Pfizer, Astellas, MSD, CUBICIN, and Novo Nordisk. He has received educational and research support from Gilead, Pfizer, Astellas, Accelerate, Novartis, Abbvie, ViiV, and MSD. Funds from European Commission/EFPIA IMI2 853966-2, IMI2 777377, H2020 847989, and ISCIII PI19/01898.

The remaining authors declare that the research was conducted in the absence of any commercial or financial relationships that could be construed as a potential conflict of interest

## Publisher’s note

All claims expressed in this article are solely those of the authors and do not necessarily represent those of their affiliated organizations, or those of the publisher, the editors and the reviewers. Any product that may be evaluated in this article, or claim that may be made by its manufacturer, is not guaranteed or endorsed by the publisher.

## Abbreviations

NAFLD, Non-alcoholic fatty liver disease; T2DM, type 2 diabetes mellitus; SGLT2i, Sodium-glucose co-transporter-2 inhibitors; GFIs, genitourinary fungal infections; NASH, non-alcoholic steatohepatitis; VHUH, Vall d’Hebron University Hospital; HE, hepatic encephalopathy; COVID-19, Coronavirus disease 2019; IQR, interquartile range; BMI, body mass index; OR, odds ratio; CI, confidence of interval; MRI, magnetic resonance imaging.
