# Supplementary material for: Risk of infections in patients with NAFLD and Type 2 Diabetes under treatment with SGLT2 inhibitors and relationship with liver outcomes: A retrospective case-control study
Source: Front Endocrinol (Lausanne). 2022 Aug 24;13:945626. doi: 10.3389/fendo.2022.945626 (PMC9449723; doi:10.3389/fendo.2022.945626)
Supplement: Supplementary file 1 [file Table_1.docx]

**Supplementary material**

**Supplementary Table 1. Test of proportional hazards assumption for the multivariable analysis of risk factors for overall infections**

| **Variable** | **rho** | **Prob>chi2** |
| --- | --- | --- |
| SGLT2i | 0.01 | 0.90 |
| HbA1c | -0.03 | 0.79 |
| Obesity† | -0.22 | 0.14 |
| Platelets | 0.11 | 0.43 |
| Age | 0.11 | 0.44 |
| Sex (male) | -0.006 | 0.96 |

HbA1c: glycosylated hemoglobin; SGLT2i: Sodium-glucose co-transporter-2 inhibitors †(BMI >30 kg/m^2^)
